# The impact of epidermolysis bullosa on quality of life and mental health

**DOI:** 10.1007/s44192-025-00209-2

**Published:** 2025-06-03

**Authors:** Guilherme Martins Freitas, Louise Hernandes Claure, Fabiana Antunes de Andrade, Kátia Sheylla Malta Purim

**Affiliations:** Department of Health Sciences and Medical School, Positivo University, Curitiba, Paraná Brazil

**Keywords:** Rare diseases, Epidermolysis bullosa, Mental health, Quality of life, Interdisciplinary studies, Health-related quality of life (HRQoL)

## Abstract

**Introduction:**

Epidermolysis bullosa (EB) is a rare, chronic, incurable, multisystemic genetic disorder characterized by skin fragility, resulting in blistering from minimal trauma or spontaneously. Despite its physical impact, little is known about the mental health and quality of life (QoL) of those affected. This study aims to investigate these aspects in individuals with EB.

**Methods:**

A cross-sectional study was conducted in Brazil using a self-administered questionnaire and a convenience sample. Data on clinical-epidemiological characteristics, QoL (DLQI-BRA and QoLEB), and depression indicators (PHQ-9) were collected. Descriptive statistics and the Chi-square test were used, with a significance level of p < 0.05.

**Results:**

The sample consisted of 31 patients, predominantly women (89.3%), with a mean age of 33.32 ± 11.3 years. The recessive dystrophic subtype was most common (54.8%), and depressive symptoms were present in 71%. PHQ-9 results revealed 29% had “mild depression,” 16.1% “moderate depression,” 22.6% “moderately severe depression,” and 3.2% “severe depression.” QoLEB indicated significant impairment in functional domains, while DLQI showed that 45.2% of patients experienced severe QoL impact. The most affected domains were leisure, symptoms and feelings, and daily activities.

**Conclusion:**

The findings highlight the substantial impact of EB on mental health, QoL, and personal relationships. These results reinforce the need for early mental health screening and the integration of multidisciplinary care to optimize patient outcomes. Implementing structured psychological support, along with comprehensive dermatological and medical management, could mitigate the burden of the disease and enhance the overall well-being of affected individuals.

## Introduction

Epidermolysis bullosa (EB) is a chronic, hereditary, and incurable multisystemic disorder characterized by mucocutaneous fragility that generates localized or generalized bullous lesions triggered by minimal trauma or that may arise spontaneously [1].

The EB complex encompasses over 30 recognized subtypes, grouped into four major groups based on the layer of the skin in which the lesions develop and underlying molecular abnormalities. The categories include EB simplex (EBS), junctional EB (JEB), dystrophic EB (DEB) and Kindler EB (KEB) [2]. Mutations have been found in several genes responsible for encoding essential proteins that maintain the structural integrity of the epidermis, basal layer, and dermis in patients with EB [1, 2]. The phenotype, severity, and prognosis are influenced by the mutated gene, disease subtype, and the patient’s overall health [1, 2].

EB is classified as a rare disease, with an estimated 500,000 individuals affected worldwide. In Brazil, although epidemiological studies are non-existing, there are 1027 cases of EB registered on the website of DEBRA Brazil [3], a Brazilian association dedicated to supporting individuals with the condition.

The clinical presentation ranges from localized palmoplantar blisters, often triggered by minor trauma and with periods of remission (localized EB simplex), to severe cases with widespread skin fragility and a heightened sensitivity to minor trauma. Complications may include recurrent infections, sepsis, pseudodactyly, and involvement of multiple systems such as the dental, nutritional, gastrointestinal, musculoskeletal, renal, and cardiovascular systems, as well as an increased risk of skin cancer [2–4].

While the physical consequences of EB are well-documented, its psychological impact remains poorly understood, particularly in underrepresented populations such as Brazilian patients. The lack of national studies assessing mental health and quality of life in individuals with EB underscores the need for further investigation into these aspects to improve patient care and guide intervention strategies.

Coping with chronic diseases can be challenging for patients. The pain and discomfort caused by skin fragility in EB can lead to adversity, negative emotions, social stigma, physical limitations, and acts of discrimination. The potential coexistence of depression, anxiety, and behavioral disorders should be considered, as they interfere with the patient's quality of life and hinder therapeutic strategies, thus worsening the clinical condition, accelerating, and aggravating the progression of EB [5].

Depression, the most prevalent mental disorder globally, affects more than 300 million people, according to the Pan American Health Organization (PAHO) [6]. A Systematic review by Tang et al. [7] has shown a link between recessive EB dystrophic and psychiatric conditions, such as anxiety and major depressive disorder (MDD). This study investigates the impact of epidermolysis bullosa on mental health and quality of life.

To comprehensively assess the impact of EB on patients’ quality of life and mental health, this study employed three validated instruments. The QoLEB is a disease-specific tool designed to evaluate quality of life in individuals with EB, providing targeted insights into their unique challenges. The DLQI-BRA is widely used in dermatology to assess the impact of skin diseases on daily life, ensuring comparability with other dermatological conditions. The PHQ-9, a well-established screening tool for depression, was included to measure the prevalence and severity of depressive symptoms in this population. Together, these instruments offer a multidimensional understanding of the physical, emotional, and psychological burden of EB.

## Methods

A cross-sectional study was conducted from April 2023 to December 2024, using an online questionnaire structured on Google Forms. The study was approved by the Research Ethics Committee of Positivo University (CAAE 69220323.0.0000.0093).

The recruitment process began with an active search for EB-related associations through websites and social media platforms. After identifying key organizations, we established initial contact with those who responded, explaining the objectives of the study and requesting their collaboration. With the support of these first associations, we gradually expanded our network, obtaining additional contacts and reaching out via email invitations to other patient groups. Recruitment strategies included announcements on social media, direct email communication, and distribution of study information within patient support groups.

Participation was voluntary, the research was conducted following the guidelines of the Research Ethics Committee of Positivo University, ensuring compliance with ethical standards, and all authors have approved the final version of the manuscript.

A convenience sample was used, and adults with a confirmed diagnosis of EB were included, with informed consent. Patients with organic neurological disorders, a history of drug abuse, communication impairments or patients regularly treated with drugs such as antidepressants or anticonvulsants were excluded.

Clinical and demographic characteristics collected were age, sex, marital status, education, family income, support network (parents, family, friends, associations, physician, etc.), time since diagnosis (in years), and type of EB [EB simplex (EBS), junctional EB (JEB), recessive EB dystrophic (REB), and dominant EB dystrophic (DEB), and Kindler EB (KEB)].

To assess quality of life, the Dermatology Life Quality Index (DLQI-BRA), a validated instrument for dermatological diseases in Brazilian Portuguese, was used. The DLQI-BRA contains ten questions and the score for each domain ranges from 0 to 3 points; thus, the total score ranges from 0 to 30, with higher scores indicating a greater degree of disability [8]. The scores are interpreted as with no impact of the patient's quality of life (0–1); little (2–5), medium (6–10), high (11–20) or very high impact (21–30).

Mental health and depression indicators were assessed using the PHQ-9 scale or Patient Health Questionnaire-9 [9]. This scale was developed based on the nine symptoms for an episode of major depression disorder (Depressed mood, anhedonia, insomnia or hipersomnia, fatigue or lack of energy, change in appetite or weight, feelings of guilt or worthlessness, concentration difficulties, feeling sluggish or restless and suicidal thoughts) described in the Diagnostic and Statistical Manual of Mental Disorders (DSM-5) [10]. It consists of nine questions (one for each symptom) and was translated into Portuguese by Brazilian psychiatrists with the reverse translation by one of the original authors. This version is available online (http://www.phqscreeners.com, accessed on 06/11/2024). The score for each domain ranges from 0 to 3 points; resulting in a total score between 0 and 27. Through the score, it is possible to assess the presence of depressive symptoms according to the levels of severity, being classified as minimal depression (1–4), mild depression (5 to 9), moderate depression (10 to 14), moderately severe depression (15 to 19), or severe depression (20 to 27 points).

To understand more specific aspects of the impact that epidermolysis bullosa has on patients’ quality of life, the Quality of Life Assessment in Epidermolysis Bullosa (QoLEB) scale [11], validated in Brazilian Portuguese, was applied. This instrument consists of 17 questions, with the score for each domain ranging from 0 to 3 points; thus, the total score varies between 0 and 51, with higher scores indicating a greater degree of impact.

Data were organized in an Excel^®^ spreadsheet and analyzed using IBM SPSS Statistics v.29.0.0 program. For the descriptive analysis of quantitative variables, the mean, standard deviation, median, minimum and maximum values were presented. Categorical variables were described using absolute frequencies and percentages.

For statistical purposes, the most common disease subtypes—DEB Dystrophic Dominant Epidermolysis Bullosa, EBS Epidermolysis Bullosa Simplex and REB Recessive Dystrophic Epidermolysis Bullosa—were compared concerning the scores from the QoLEB, DLQI, and PHQ-9 instruments, using the non-parametric Kruskal–Wallis’ test and the Dunn's post-hoc test. Internal consistency of the PHQ-9, QoLEB, and DLQI questionnaires was assessed by Cronbach’s alpha coefficient, with values between 0.7 and 0.95 indicating good internal consistency [16]. A p-value of < 0.05 was considered statistically significant. For pairwise subtype comparisons, p values were adjusted by Bonferroni.

## Results

Responses were obtained from 35 patients with epidermolysis bullosa (EB), and after applying the inclusion criteria, 31 of them were eligible and comprised the sample. All participants completed the QoLEB, DLQI, and PHQ-9 questionnaires. Among the 31 participants, 83.9% were female and 16.1% male, with a mean age of 33.32 ± 11.31, ranging from 19 to 60 years old. Most patients were single (67.7%) and had recessive dystrophic EB (54.8%). The main support network for people with EB consisted of their family members, particularly the mother (77.1%) and father (57.1%) most often playing this role (Table 1).

**Table 1:** General characteristics of the patients with epidermolysis bullosa studied (N = 31)

General characteristics (N = 31)	N	%
Gender		
Female	26	(83.9)
Male	5	(16.1)
Age, years		
Mean ± SD	33.32	± 11.31
Marital status		
Married	8	(25.8)
Divorced	1	(3.2)
Single	21	(67.7)
Common-law marriage	1	(3.2)
Education		
Incomplete elementary school	1	(3.2)
Complete high school	10	(32.3)
Incomplete high school	2	(6.5)
Complete higher education	7	(22.6)
Incomplete higher education	4	(12.9)
Graduate degree	7	(22.6)
Family income		
Up to 1 minimum wage	4	(12.9)
Up to 2 minimum wages	10	(32.3)
Up to 3 minimum wages	5	(16.1)
Up to 4 minimum wages	2	(6.5)
Up to 5 minimum wages	3	(9.7)
> 5 minimum wages	7	(22.6)
Age at diagnosis		
> 10 years old	8	(25.8)
1–5 years old	1	(3.2)
5–10 years old	2	(6.5)
Up to 1 year	20	(64.5)
Type of EB		
Dominant dystrophic EB (DEB)	6	(19.4)
Recessive dystrophic EB (REB)	17	(54.8)
Junctional EB (JEB)	2	(6.5)
Kindler EB (KEB)	1	(3.2)
EB Simplex (EBS)	5	(16.1)

SD: Standard deviation

Most participants (64.5%) received an EB diagnosis within the first year of life. Early diagnosis is crucial for initiating appropriate care, implementing preventive measures, and improving long-term health outcomes. Additionally, 45.2% reported a household income of up to two minimum wages. Given the chronic nature of EB, access to specialized care and treatment, including high-cost wound dressings, may be a challenge for patients in lower socioeconomic conditions. While our study did not perform a specific analysis of these factors, future research should explore their potential impact on quality of life and mental health.

Higher scores in the QoLEB and DLQI indicate greater impairment in quality of life, while higher PHQ-9 scores reflect increased severity of depressive symptoms. The QoLEB measures the impact of EB-specific factors on daily activities, with scores ranging from 0 (no impact) to 51 (maximum impact). The DLQI is a general dermatology-related quality of life index, with scores from 0 to 30, where higher values denote greater disability. The PHQ-9 is a validated depression screening tool, with scores from 0 to 27, classified as minimal (1–4), mild (5–9), moderate (10–14), moderately severe (15–9), and severe depression (20–27).

The scores of the QoLEB, DLQI, and PHQ-9 instruments indicated that the disease negatively impacts the patient's quality of life. (Tables 2 and 3).

**Table 2:** Scores of the QoLEB, DLQI and PHQ-9 instruments in patients with epidermolysis bullosa (N = 31)

Variable	Score variation	n	Mean	Standard deviation	Median	Minimum	Maximum
QoLEB*							
Functional domain	0 to 36	31	11.0	6.6	10	2	27
Emotional domain	0 to 15	31	6.8	3.3	7	0	13
General	0 to 51	31	17.9	9.2	19	2	40
DLQI**							
Symptoms and feelings	0 to 6	31	2.7	1.7	3	0	6
Daily activities	0 to 6	31	1.9	1.7	2	0	6
Leisure	0 to 6	31	2.9	1.9	3	0	6
Work and school	0 to 3	31	1.0	1.4	0	0	3
Personal relationship	0 to 6	31	1.7	1.7	1	0	6
Treatment	0 to 3	31	0.7	1.1	0	0	3
General	0 to 30	31	10.9	7.7	9	0	29
PHQ-9***							
General	0 to 27	31	9.2	6.4	8	0	22

QoLEB, Quality of Life Assessment in Epidermolysis Bullosa;

DLQI, Dermatology Life Quality Index;

PHQ-9, Patient Health Questionnaire;

^*^The higher the score, the worse the quality of life concerning health

^**^The higher the score, the greater the impact of the disease on the quality of life

^***^The higher the score, the greater the intensity of depression

**Table 3:** Disease subtypes and DLQI and PHQ-9 questionnaire scores in patients with Epidermolysis Bullosa (N = 31)

Variable	Classification	n	%
DLQI-classification	No impact on patient’s life	1	3.2
	Little impact on patient’s life	5	16.1
	Medium impact on patient’s life	10	32.3
	High impact on patient’s life	9	29.0
	Very high impact on patient’s life	6	19.4
DLQI—cutoff point of 10	≤ 10 (Not severely impacted by the disease)	17	54.8
	> 10 (Life severely impacted by the disease)	14	45.2
PHQ-9 classification	Minimal depression	9	29.0
	Mild	9	29.0
	Moderate	5	16.1
	Moderately severe	7	22.6
	Severe	1	3.2

DLQI, Dermatology Life Quality Index; PHQ-9, Patient Health Questionnaire

In our sample, the mean DLQI score was 10.9 ± 7.7, with 45.2% of participants exceeding the cutoff score of 10, indicating severe impairment in QoL. The most affected domains included leisure, symptoms and feelings, and daily activities. These findings highlight the substantial burden of EB on patients’ daily lives, which will be further explored in the discussion.

The mean PHQ-9 score was 9.2 ± 6.4, showing that 71% of participants experienced at least mild depressive symptoms. The QoLEB results showed a mean score of 17.9 ± 9.2, reinforcing the substantial burden of EB on daily functioning. These findings highlight the importance of evaluating both dermatological and psychological aspects in EB management.

The subtypes of the disease that had the highest frequencies (DEB, REB and EBS) were compared concerning the scores of the QoLEB, DLQI, and PHQ-9 instruments. A statistically significant difference was observed in the DLQI personal relationships domain among EB subtypes (p = 0.045). However, given the small sample size, these findings should be interpreted with caution, as our study was not designed to perform robust subgroup comparisons (Table 4).

**Table 4:** Scores of the QoLEB, DLQI, and PHQ-9 instruments for each of the EBD, EBR, and EBS subtypes

Variable	Disease subtype	n	Mean	Standard deviation	Median	Minimum	Maximum	p*
QoLEB—functional domain	DEB	6	10.7	5.2	10.5	4	19	
	REB	17	11.3	5.7	10	4	21	
	EBS	5	12.8	11.4	11	2	27	0.981
QoLEB—emotional domain	DEB	6	7.8	3.0	7.5	3	11	
	REB	17	6.9	3.0	7	2	12	
	EBS	5	6.6	5.1	8	0	13	0.839
QoLEB—general	DEB	6	18.5	7.7	18	10	30	
	REB	17	18.2	7.6	19	7	30	
	EBS	5	19.4	16.3	19	2	40	0.997
DLQI—symptoms and feelings	DEB	6	2.8	0.8	3	2	4	
	REB	17	2.6	1.5	2	0	5	
	EBS	5	3.6	2.6	4	0	6	0.604
DLQI—daily activities	DEB	6	1.7	1.4	1.5	0	4	
	REB	17	1.5	1.3	2	0	3	
	EBS	5	3.6	2.9	5	0	6	0.334
DLQI—leisure	DEB	6	3.2	1.5	3.5	1	5	
	REB	17	2.5	1.8	3	0	5	
	EBS	5	4.2	2.7	6	0	6	0.264
DLQI—work and school	DEB	6	2	1.5	3	0	3	
	REB	17	0.7	1.3	0	0	3	
	EBS	5	1.2	1.6	0	0	3	0.173
DLQI—personal relationships	DEB	6	2.5	1.4	2.5	1	4	
	REB	17	1.2	1.4	1	0	5	
	EBS	5	3.2	2.3	3	1	6	0.045**
DLQI—treatment	DEB	6	0.8	1.0	0.5	0	2	
	REB	17	0.6	1.0	0	0	3	
	EBS	5	1.2	1.6	0	0	3	0.754
DLQI—total	DEB	6	13.0	5.6	13	7	20	
	REB	17	9.3	6.4	10	0	21	
	EBS	5	17	12.7	19	1	29	0.282
PHQ-9 total	DEB	6	11.5	4.8	12	5	17	
	REB	17	8.9	6.6	8	0	22	
	EBS	5	9.6	8.3	12	0	19	0.611

QoLEB, Quality of Life Assessment in Epidermolysis Bullosa; PHQ-9, Patient Health Questionnaire

p, 0.185; (p-values corrected by Bonferroni)

DEB, Dominant Dystrophic Epidermolysis Bullosa

EBS, Epidermolysis Bullosa Simplex

REB, Recessive Dystrophic Epidermolysis Bullosa

^*^Non-parametric Kruskal–Wallis’ test and Dunn’s post-hoc test, p < 0.05

^**^Two-by-two subtype comparisons for DLQI-personal relationships: DEB x REB: p = 0.185; DEB x REB:

## Discussion

Epidermolysis bullosa remains a relatively understudied disease. Our comprehensive search of MEDLINE, Latindex, and BVS databases revealed this as one of the first, if not the first, Brazilian study to extensively examine psychological aspects and quality of life in individuals with EB, using one of the largest sample sizes worldwide.

The present study highlights a group of individuals diagnosed with the same rare and challenging disease, although the clinical picture varies between subtypes. Notably, 64.5% of patients were diagnosed within the first year of life. This finding underscores the importance of physicians being well-prepared to make an early diagnosis, as early interventions can help mitigate serious complications. Furthermore, the variability in clinical presentation across subtypes reinforces the need for tailored, multidisciplinary care to address the specific needs of each patient and improve their overall quality of life. The significant role of the mother and/or father in the support network suggests that family dynamics are likely both influenced by and a determinant of the health-disease process. Family affection is a positive environmental factor that helps patients to better cope with the disease [12].

The financial burden of EB treatment is a significant concern, as specialized wound care products, such as atraumatic dressings and topical medications, are often expensive and not always available through public healthcare systems. In our study, nearly half of the participants reported a family income of up to two minimum wages, which may limit access to these essential resources. Socioeconomic disparities could contribute to variations in treatment adherence, disease progression, and overall quality of life. These findings reinforce the need for health policies aimed at ensuring equitable access to EB treatments.

Our cohort consisted predominantly of female participants (83.9%) and individuals with REB (54.8%). One possible explanation is that individuals with REB, due to the severity of their condition, are more frequently engaged with healthcare services, making them more likely to be reached during recruitment. Additionally, differences in healthcare-seeking behavior between sexes, observed in various medical conditions, may have influenced the composition of our sample. This pattern aligns with previous studies suggesting that men tend to perceive themselves as autonomous and in control, often avoiding healthcare services, downplaying discussions about pain, and hesitating to acknowledge its connection with psychological well-being [13]. These demographic imbalances should be considered when interpreting the findings.

EB treatment involves medication, specialized skin care, and other demands, which can cause suffering, stress, fatigue, and conflicts for the patient and their family. Understanding this dynamic is essential for planning medical, psychological, and social interventions that are more assertive for each patient's clinical conditions. Genetic counseling is also recommended since even with a low calculated risk, EB may be present in other family members [5].

In the present study, 45.2% of participants scored above the DLQI cutoff of 10, indicating severe impairment in quality of life. The domains most affected by the disease were leisure, symptoms and feelings, and daily activities. The continuous need for wound care, risk of infections, and physical discomfort associated with skin fragility likely contribute to emotional distress and social isolation. A study by Korte et al. [14] suggests that these patients often experience a significant impact on various life domains, including reduced mobility due to painful blisters on the hands and feet, which affect their ability to work and engage in hobbies for example. Additionally, some patients reported feeling socially excluded due to the limitations imposed by EB, further compounded by the invisibility of their symptoms.

In line with previous literature [12], our study found that EB patients tend to prioritize aspects less directly affected by the disease, such as family, school, and personal relationships. This prioritization likely serves as a physical and psychological coping mechanism against the challenges posed by the condition.

According to the Pan American Health Organization (PAHO) [6], the global prevalence of depressive symptoms in the general population is estimated at 3.8%. In contrast, the prevalence observed in our study was significantly higher at 71%, encompassing varying degrees of depression: 29% classified as mild, 16.1% as moderate, 22.6% as moderately severe, and 3.2% as severe depression.

This stark contrast suggests that individuals with EB may be at substantially higher risk for depressive disorders compared to the general population. The chronic nature of EB, persistent pain, physical limitations, and social stigma are all factors that may contribute to this increased vulnerability. Given these findings, mental health assessment should be an integral part of EB management, ensuring that affected individuals receive timely psychological support and intervention.

Notably, the QoLEB instrument indicated greater impairment in the functional domain than in the emotional domain, highlighting that while physical limitations are substantial, psychological adjustment varies among individuals. Although many participants reported a moderate-to-severe impact on quality of life (DLQI ≥ 10), 29% presented with minimal depressive symptoms and 29% with mild depressive symptoms. This suggests that while some patients develop adaptation mechanisms, factors such as disease acceptance and social support may play a role in alleviating depressive symptoms in certain cases.

Although a statistically significant difference was not found in most comparisons between EB subtypes, the small sample size limits our ability to detect potential differences. Therefore, we caution against overinterpreting these trends. The observed variation in QoL scores may reflect individual differences in symptom perception rather than true subtype-related disparities.

The identification of depressive symptoms, reduced quality of life, and functional impairments observed in this study aligns with findings reported in the literature [15]. These results underscore the importance of implementing strategies to provide emotional support, reduce stigma, and enhance social integration. Community-based interventions and telehealth services play a crucial role in this process, as patient associations, peer support groups, and psychological counseling can help individuals with EB develop coping mechanisms and reduce feelings of isolation.

Additionally, educational campaigns targeting healthcare professionals, caregivers, and the general public can be valuable in raising awareness about the disease, promoting early diagnosis, and fostering a more inclusive social environment. Addressing misconceptions and increasing knowledge about EB may reduce stigma and improve the quality of interactions between patients, families, and the broader community.

## Limitations

This study has some limitations. First, the small sample size reduces the statistical power to detect significant differences between EB subtypes, limiting our ability to draw definitive conclusions about subgroup variations. While trends were observed, the study was not designed for robust subgroup comparisons.

Second, data collection relied on self-reported questionnaires, which may introduce recall bias and subjective interpretation. Although validated instruments were used, incorporating clinician assessments or caregiver perspectives in future studies could enhance the accuracy of findings.

Third, the over-representation of female participants (83.9%) and individuals with REB (54.8%) in our sample may impact the generalizability of our findings. Future studies with larger and more balanced samples are needed to ensure broader representation of all EB subtypes and male patients.

Lastly, the cross-sectional design provides only a snapshot of mental health and quality of life in EB patients, preventing an assessment of changes over time. Longitudinal studies are needed to track psychological outcomes and identify key intervention points. Future research should also include larger and more diverse samples to improve generalizability and better understand the psychological burden of EB across different populations.

## Conclusion

This study highlights the substantial impact of EB on mental health and quality of life, with a high prevalence of depressive symptoms (71%) and significant impairment in daily activities. These findings emphasize the critical need for routine psychiatric screening and a multidisciplinary approach, integrating dermatological, psychological, and social support to optimize patient care.

Considering the chronic and disabling nature of EB, expanding access to mental health resources, community-based interventions, and financial support for specialized treatments is essential to improving patient outcomes. Given the small sample size and cross-sectional nature of this study, future research with larger cohorts and longitudinal follow-up is needed to better understand the psychological burden of EB over time and evaluate the effectiveness of targeted interventions. Health policies that facilitate equitable access to treatment and psychosocial support could play a key role in reducing the disease burden and improving patients’ quality of life.

## Data Availability

The datasets generated and analysed during the current study are not publicly available due to restrictions based on informed consent. Participants did not agree to have their data shared publicly, but the datasets are available from the corresponding author on reasonable request.
